# Volunteer Surgeons

**Published:** 1862-05

**Authors:** 


VOLUNTEER SURGEONS.—Dr. Sanford B. Hunt, formerly Professor of Anatomy and Physiology in the University of Buffalo, and editor of the *Buffalo Medical Journal*, and Dr. Charles Winne, Surgeon to the Hospital of the Sisters of Charity, have received commissions from Major-General Morgan, as members of the corps of Volunteer Surgeons, now being organized. These distinguished members of the profession have the highest qualifications for the position, and should their assistance be required, will render efficient service.

